# 2,6-Anhydro-1,3-di-*O*-benzyl-d-mannitol

**DOI:** 10.1107/S1600536811022306

**Published:** 2011-06-18

**Authors:** Edmilson Clarindo de Siqueira, Bogdan Doboszewski, James McGarrah, Alexander Y. Nazarenko

**Affiliations:** a Departamento de Química, Universidade Federal Rural de Pernambuco, 52171-900 Recife, PE, Brazil; b Department of Chemistry, State University of New York, College at Geneseo, 1 College Circle, Geneseo, NY 14454, USA; c Chemistry Department, State University of New York, College at Buffalo, 1300 Elmwood Avenue, Buffalo, NY 14222-1095, USA

## Abstract

In the title compound, C_20_H_24_O_5_, the six-membered pyran­ose ring adopts a chair conformation. The dihedral angle between the planes of the phenyl groups of the benzyl substituents is 63.1°. Two types of inter­molecular O—H⋯O hydrogen bonds lead to the formation of infinite chains along the *b* axis. Only weak C—H⋯O contacts exist between neighboring chains.

## Related literature

For syntheses of this and similar compounds, see: Barker (1970 ▶); Doboszewski (1997 ▶, 2009 ▶); Doboszewski & de Siqueria (2010 ▶); Hartman (1970*a* ▶,*b* ▶). For related structures, see: Boeyens *et al.* (1983 ▶); Doboszewski & Nazarenko (2003 ▶); Guiry *et al.* (2008 ▶); Hong *et al.* (2005 ▶); Vidra *et al.* (1982 ▶). For conformations of six-membered rings, see: Schwarz (1973 ▶); Cremer & Pople (1975 ▶); Boeyens & Dobson (1987 ▶). For hydrogen bonding in carbohydrate chemistry, see Gilli & Gilli (2009 ▶); Desiraju & Steiner (1999 ▶); Jeffrey (1997 ▶), and references therein.
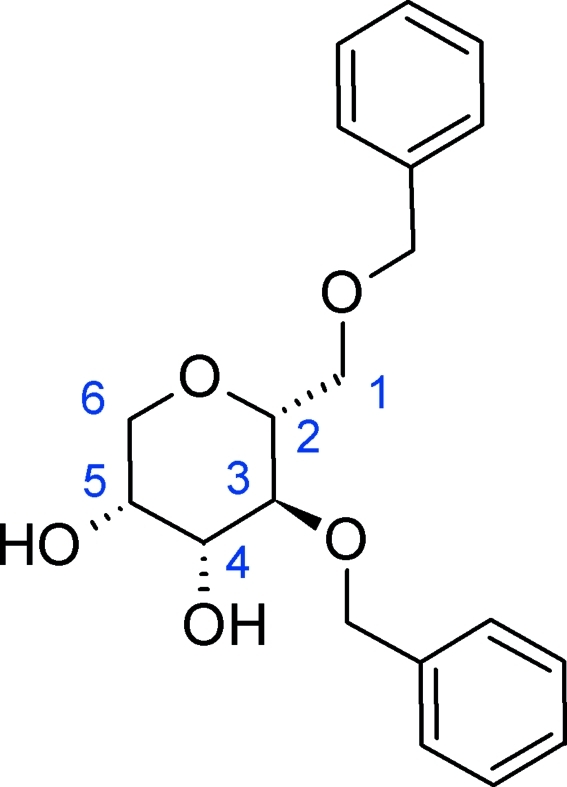


## Experimental

### 

#### Crystal data

C_20_H_24_O_5_
*M* *_r_* = 344.39
Monoclinic, 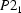

*a* = 5.6584 (10) Å
*b* = 7.9610 (12) Å
*c* = 19.808 (4) Å
β = 91.968 (6)°
*V* = 891.8 (3) Å^3^
*Z* = 2
Mo *K*α radiation
μ = 0.09 mm^−1^
*T* = 200 K
0.6 × 0.4 × 0.05 mm

#### Data collection

Bruker SMART X2S diffractometer
Absorption correction: multi-scan (*SADABS*; Sheldrick, 2008*b* ▶) *T*_min_ = 0.91, *T*_max_ = 0.98
8624 measured reflections
1695 independent reflections
1458 reflections with *I* > 2σ(*I*)
*R*_int_ = 0.052

#### Refinement

*R*[*F* ^2^ > 2σ(*F* ^2^)] = 0.035
*wR*(*F* ^2^) = 0.082
*S* = 0.99
1695 reflections
228 parameters
1 restraint
H-atom parameters constrained
Δρ_max_ = 0.19 e Å^−3^
Δρ_min_ = −0.14 e Å^−3^

### 

Data collection: *GIS* (Bruker, 2010 ▶); cell refinement: *APEX2* (Bruker, 2010 ▶) and *SAINT* (Bruker, 2009 ▶); data reduction: *SAINT* and *XPREP* in *SHELXTL* (Sheldrick, 2008*a* ▶); program(s) used to solve structure: *SHELXS97* (Sheldrick, 2008*a* ▶); program(s) used to refine structure: *SHELXL97* (Sheldrick, 2008*a* ▶); molecular graphics: *ORTEP-3 for Windows* (Farrugia, 1999 ▶) and *Mercury* (Macrae *et al.*, 2008 ▶); software used to prepare material for publication: *PLATON* (Spek, 2009 ▶).

## Supplementary Material

Crystal structure: contains datablock(s) I, global. DOI: 10.1107/S1600536811022306/zl2379sup1.cif

Structure factors: contains datablock(s) I. DOI: 10.1107/S1600536811022306/zl2379Isup2.hkl

Additional supplementary materials:  crystallographic information; 3D view; checkCIF report

**Fig. 5.**
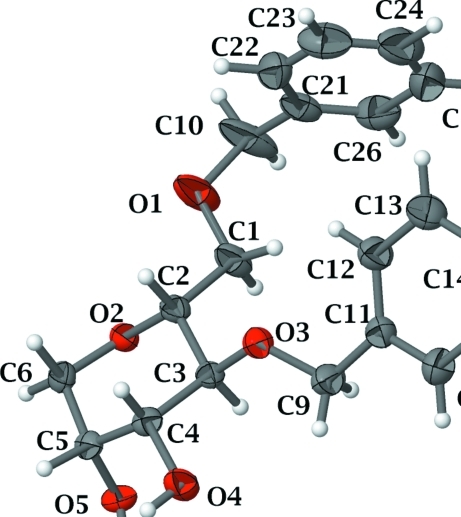
Additional figure.

Enhanced figure: interactive version of Fig. 5

## Figures and Tables

**Table 1:** Hydrogen-bond geometry (Å, °)

*D*—H⋯*A*	*D*—H	H⋯*A*	*D*⋯*A*	*D*—H⋯*A*
O4—H4⋯O5^i^	0.84	1.95	2.789 (2)	175
O5—H5⋯O2^i^	0.84	1.98	2.812 (2)	169
C6—H6*B*⋯O5^ii^	0.99	2.54	3.461 (3)	155

Symmetry codes: (i) 
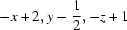
; (ii) 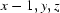.

**Fig. 1.**
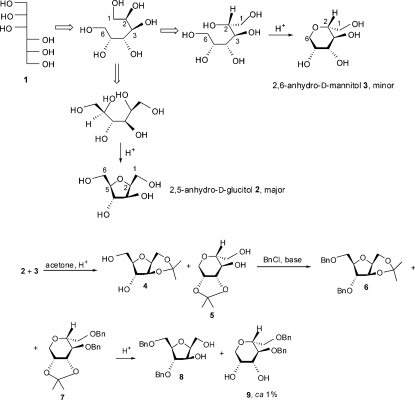
Two possibilities of dehydration of D-mannitol relevant to the synthesis of 1,5-anhydro-D-mannitol and formation of the dibenzyl ethers.

**Fig. 2.**
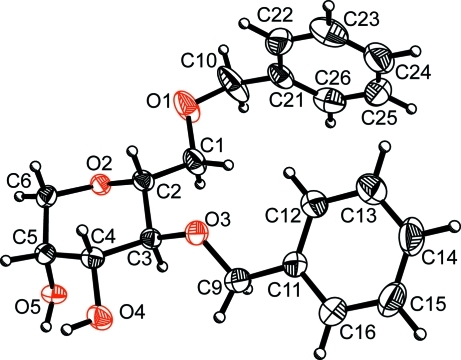
ORTEP view of 1,5-anhydro-4,6-di-O-benzyl-D-mannitol with displacement ellipsoids drawn at the 50% probability level.

**Fig. 3.**
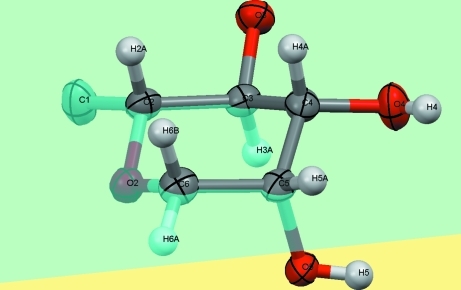
Chair conformation of the six-membered ring.

**Fig. 4.**
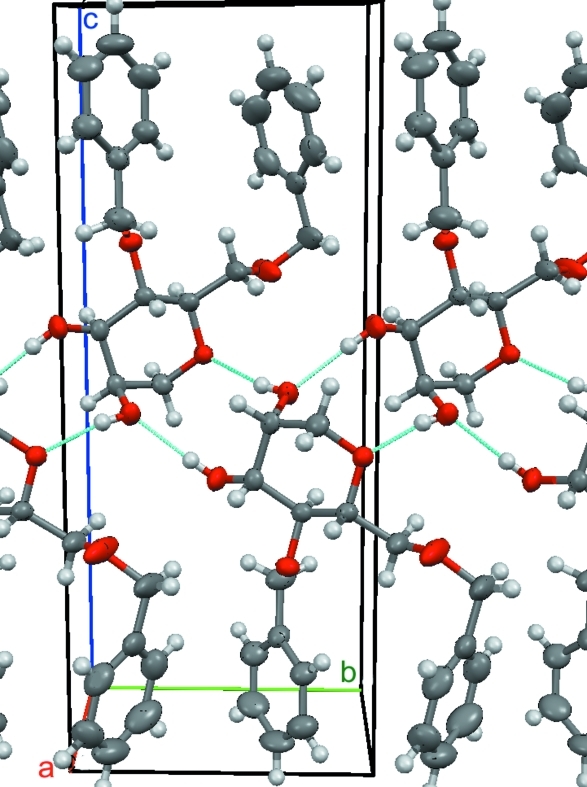
Infinitive chain of 2,6-anhydro-1,3-di-O-benzyl-D-mannitol molecules along the b axis. View along the a axis.

## supplementary crystallographic information

### Comment

A target compound of our (BD and ECS) synthetic research, 2,5-anhydro-D-glucitol (compound 2 in Fig. 1) is technically a β-C-glycoside of *D*-arabinofuranose. We prepared it in its protected form 4 starting from 2,3,5-tri-*O*-benzyl-*D*-arabinofuranosyl chloride or bromide (Doboszewski, 1997, 2009). The structure of 4 was confirmed by X-ray crystallography (Doboszewski & Nazarenko, 2003). Since this procedure furnished low and variable yields, we focused our attention on an alternative method, *i.e.* acid-catalyzed dehydration of D-mannitol 1 (Barker, 1970; Hartman, 1970*a*,*b*; Doboszewski & de Siqueria, 2010). The original patented procedure (Hartman, 1970*a*,*b*) was modified by using vacuum-dry chromatography to isolate the acetonide 4, which was subsequently used to obtain the corresponding di-*O*-benzyl derivative 8 (Doboszewski, 1997). During the synthesis of 8 at a *ca* 30 g scale (see Fig. 1) we have noticed the presence of a minor byproduct which is more polar than the expected 8. This compound was formed in a very low yield (*ca* 1%) and its ^1^H NMR spectrum was practically intractable and showed an aromatic:aliphatic H atom ratio of 1:1.4. Using single-crystal X-ray diffraction (this present study) it was identified as 2,6-anhydro-1,3-di-*O*-benzyl-D-mannitol 9. Evidently, the main cyclization route to form 2,5-anhydro-D-glucitol 2 was accompanied by a minor pathway to form 3 together with other dehydration products (Barker, 1970). Both acetonides 4 and 5 migrated jointly during chromatography, but become separable after transformation into the corresponding di-benzyl ethers 8 and 9, respectively (Fig. 1).

The absolute structure of the title compound is known from the synthetic route which does not affect stereogenic atoms of the starting D-mannitol. In the crystal structure of title compound (Fig. 2), all bond lengths and bond angles have standard dimensions. The high flexibility of the oxymethylene fragment results in elongated thermal ellipsoids of atoms O1 and C10.

The six-membered phenyl rings are flat within 0.01 Å. Fig. 3 shows that the pyranose ring adopts a chair conformation (Schwarz, 1973) with atoms C1, C2, C5, and C6 being within 0.01 Å from their mean plane, and atoms O1 and C4 at a distances of 0.68 and 0.64 Å. A quantitative analysis of the ring conformations was performed using the method of Cremer and Pople (Cremer & Pople, 1975; Boeyens & Dobson, 1987) for the calculation of parameters of puckering. The polar parameters for the pyranose ring are Q = 0.576 (2) Å, Θ = 175.8 (2)°, and Φ = 207 (3)°. These suggest a chair conformation (ideal Θ = 0 or 180°) only slightly distorted towards half-chair (Θ = 130°, Φ = 210°). There are four compounds reported in Cambridge Structure Database with the same motif: 1,5-anhydro-DL-galactitol (refcode ANGALA10, Vidra *et al.*, 1982) 1,5-anhydro-D-glucitol (CELTUI, Boeyens *et al.*, 1983), (+)-ethyl-3-(acetoxy)-4,5-dihydroxytetrahydro-2*H*-pyran-2-carboxylate (FIQWAE, Hong *et al.*, 2005) and 1-deoxy-D-lactose (XOJLUE, Guiry *et al.*, 2008). In all these structures, the six-membered ring has a chair conformation.

Two hydroxy groups and an O atom of the pyranose ring form a system of O—H··· O hydrogen bonds that leads to the formation of an infinitive chain along the *b* axis (Table 1, Fig. 4). These hydrogen bonds of intermediate strength (Gilli & Gilli, 2009) result in a decrease of the O—H stretching vibrations frequency from the theoretical 3500 cm^-1^ for a "free" OH group to 3330 cm^-1^.

Only weak C—H···O (Table 1) contacts exist between neighboring chains. Similar bonds were observed in various carbohydrates (Desiraju & Steiner, 1999). A short intramolecular contact between oxygen O3 and H atom H1A of neighboring methylene group may additionally stabilize the conformation of the molecule.

### Experimental

Crystals of the title compound were obtained as a side product of dehydration of D-mannitol (Fig. 1) in the form of thin plates (m.p. 454 (3) K) by spontaneous crystallization after chromatographic separation using a gradient of ethylacetate in hexane. A suitable crystal was cut out of a larger plate. Data collection was limited to θ = 25° because of the geometry of the instrument.

Exact mass MS (ESI): calc. for C_20_H_24_O_5_ +Na^+^: 367.1516; found 367.1507.

Optical rotation: α_D_ +7.4° c 2.6 (DMSO)

^1^H NMR (300 MHz, DMSO-*d*_6_): 7.37–7.25 (H aromatic, 10H), 4.86 (d, *J* = 6.3 Hz, 1H, exchangeable), 4.83 (d, *J* = 11.1 Hz, 1H), 4.65 (d, *J* = 3.6 Hz, exchangeable), 4.51 (d, *J* = 11.6 Hz, 2H), 4.44 (d, *J* = 12.1 Hz, 1H), 3.76–3.39 (unresolved, 7H), 3.27–3.21 (unresolved, 1H).

^13^C NMR: 139.02, 138.42, 128.21, 128.10, 127.63, 127.39, 127.28, 78.66, 75.98, 74.36, 73.76, 72.33,* 69.77,* 69.69,* 69.29 (* negative signals in the Attached Proton Test).

FT–IR (Nicolet 400, diamond ATR): 3330 (very strong), 3064, 3033, 2916, 2862, 1495, 1452, 1328, 1082, 1067, 890, 692, 606, 530 cm^-1^.

Raman (Raman Systems 2.0; 785 nm laser): 1603, 1466, 1342, 1278, 1202, 1174, 1004 (very strong), 945, 821, 617, 431, 186 cm^-1^.

### Refinement

The chirality of the title compound was known from the synthetic route. Therefore, Friedel pairs were treated as equivalents at data processing and were merged at refinement. Reflection 0 0 1 was obstructed by the beam stop and was omitted.

All H atoms were positioned geometrically with *U*_iso_(H) = 1.2 or 1.5*U*_eq_(C) with refined torsion angles for H4 and H5 (AFIX 147 command in *SHELXL* (Sheldrick, 2008*a*)).

### Crystal data

**Table:**

C_20_H_24_O_5_	*F*(000) = 368
*M**_r_* = 344.39	*D*_x_ = 1.283 Mg m^−^^3^
Monoclinic, *P*2_1_	Melting point: 454(3) K
Hall symbol: P 2yb	Mo *K*α radiation, λ = 0.71073 Å
*a* = 5.6584 (10) Å	Cell parameters from 2505 reflections
*b* = 7.9610 (12) Å	θ = 2.1–25.0°
*c* = 19.808 (4) Å	µ = 0.09 mm^−^^1^
β = 91.968 (6)°	*T* = 200 K
*V* = 891.8 (3) Å^3^	Plate, colourless
*Z* = 2	0.6 × 0.4 × 0.05 mm

### Data collection

**Table:**

Bruker SMART X2S diffractometer	1695 independent reflections
Radiation source: XOS X-beam microfocus source	1458 reflections with *I* > 2σ(*I*)
doubly curved silicon crystal	*R*_int_ = 0.052
ω scans	θ_max_ = 25.0°, θ_min_ = 2.1°
Absorption correction: multi-scan (*SADABS*; Sheldrick, 2008*b*)	*h* = −6→6
*T*_min_ = 0.91, *T*_max_ = 0.98	*k* = −9→9
8624 measured reflections	*l* = −23→23

### Refinement

**Table:**

Refinement on *F*^2^	Primary atom site location: structure-invariant direct methods
Least-squares matrix: full	Secondary atom site location: difference Fourier map
*R*[*F*^2^ > 2σ(*F*^2^)] = 0.035	Hydrogen site location: inferred from neighbouring sites
*wR*(*F*^2^) = 0.082	H-atom parameters constrained
*S* = 0.99	*w* = 1/[σ^2^(*F*_o_^2^) + (0.0509*P*)^2^] where *P* = (*F*_o_^2^ + 2*F*_c_^2^)/3
1695 reflections	(Δ/σ)_max_ < 0.001
228 parameters	Δρ_max_ = 0.19 e Å^−^^3^
1 restraint	Δρ_min_ = −0.14 e Å^−^^3^

### Special details

**Table:**

Geometry. All e.s.d.'s (except the e.s.d. in the dihedral angle between two l.s. planes) are estimated using the full covariance matrix. The cell e.s.d.'s are taken into account individually in the estimation of e.s.d.'s in distances, angles and torsion angles; correlations between e.s.d.'s in cell parameters are only used when they are defined by crystal symmetry. An approximate (isotropic) treatment of cell e.s.d.'s is used for estimating e.s.d.'s involving l.s. planes.
Refinement. Refinement of *F*^2^ against ALL reflections. The weighted *R*-factor *wR* and goodness of fit *S* are based on *F*^2^, conventional *R*-factors *R* are based on *F*, with *F* set to zero for negative *F*^2^. The threshold expression of *F*^2^ > σ(*F*^2^) is used only for calculating *R*-factors(gt) *etc*. and is not relevant to the choice of reflections for refinement. *R*-factors based on *F*^2^ are statistically about twice as large as those based on *F*, and *R*-factors based on ALL data will be even larger.

### Fractional atomic coordinates and isotropic or equivalent isotropic displacement parameters (Å^2^)

**Table:**

	*x*	*y*	*z*	*U*_iso_*/*U*_eq_	
O1	0.5054 (4)	0.6502 (3)	0.64947 (9)	0.0699 (7)	
O2	0.6876 (3)	0.44181 (18)	0.54580 (7)	0.0288 (4)	
O3	0.8458 (3)	0.2160 (2)	0.70085 (7)	0.0329 (4)	
O4	1.0068 (3)	−0.0091 (2)	0.59915 (8)	0.0389 (4)	
H4	0.9884	−0.0925	0.5735	0.047*	
O5	1.0291 (3)	0.2171 (2)	0.48840 (8)	0.0311 (4)	
H5	1.1187	0.1341	0.4836	0.037*	
C1	0.7134 (5)	0.5512 (3)	0.65761 (12)	0.0426 (7)	
H1A	0.7402	0.5208	0.7057	0.051*	
H1B	0.8523	0.6151	0.6427	0.051*	
C2	0.6801 (4)	0.3952 (3)	0.61547 (11)	0.0296 (5)	
H2A	0.5206	0.3471	0.6240	0.036*	
C3	0.8671 (4)	0.2607 (3)	0.63147 (10)	0.0272 (5)	
H3A	1.0284	0.3072	0.6241	0.033*	
C4	0.8223 (4)	0.1094 (3)	0.58552 (10)	0.0285 (5)	
H4A	0.6689	0.0569	0.5976	0.034*	
C5	0.8049 (4)	0.1607 (3)	0.51169 (11)	0.0275 (5)	
H5A	0.7506	0.0621	0.4839	0.033*	
C6	0.6287 (4)	0.3020 (3)	0.50228 (11)	0.0311 (5)	
H6A	0.6261	0.3397	0.4546	0.037*	
H6B	0.4687	0.2606	0.5123	0.037*	
C9	1.0627 (4)	0.1952 (4)	0.73746 (12)	0.0466 (7)	
H9A	1.1698	0.2896	0.7274	0.056*	
H9B	1.1392	0.0897	0.7233	0.056*	
C10	0.5053 (8)	0.7934 (4)	0.69327 (14)	0.0781 (12)	
H10A	0.3683	0.8656	0.6807	0.094*	
H10B	0.6509	0.8596	0.6867	0.094*	
C11	1.0226 (4)	0.1894 (3)	0.81204 (11)	0.0358 (6)	
C12	0.8296 (5)	0.2669 (4)	0.84079 (12)	0.0436 (7)	
H12A	0.7150	0.3227	0.8127	0.052*	
C13	0.8036 (5)	0.2634 (4)	0.90972 (13)	0.0538 (8)	
H13A	0.6711	0.3162	0.9289	0.065*	
C14	0.9691 (5)	0.1834 (5)	0.95082 (13)	0.0595 (9)	
H14A	0.9516	0.1822	0.9983	0.071*	
C15	1.1608 (5)	0.1049 (4)	0.92311 (13)	0.0579 (8)	
H15A	1.2745	0.0488	0.9514	0.069*	
C16	1.1858 (5)	0.1086 (4)	0.85404 (13)	0.0464 (7)	
H16A	1.3177	0.0545	0.8351	0.056*	
C21	0.4931 (5)	0.7468 (4)	0.76631 (13)	0.0437 (7)	
C22	0.3085 (5)	0.6518 (4)	0.79079 (15)	0.0546 (8)	
H22A	0.1873	0.6120	0.7605	0.066*	
C23	0.2996 (6)	0.6149 (5)	0.85879 (17)	0.0656 (9)	
H23A	0.1729	0.5496	0.8750	0.079*	
C24	0.4711 (7)	0.6713 (5)	0.90252 (15)	0.0693 (10)	
H24A	0.4632	0.6463	0.9493	0.083*	
C25	0.6537 (6)	0.7633 (5)	0.87996 (16)	0.0699 (10)	
H25A	0.7729	0.8032	0.9109	0.084*	
C26	0.6663 (5)	0.7989 (4)	0.81241 (16)	0.0553 (8)	
H26A	0.7974	0.8609	0.7970	0.066*	

### Atomic displacement parameters (Å^2^)

**Table:**

	*U*^11^	*U*^22^	*U*^33^	*U*^12^	*U*^13^	*U*^23^
O1	0.1104 (18)	0.0642 (16)	0.0345 (10)	0.0575 (15)	−0.0080 (10)	−0.0070 (10)
O2	0.0307 (9)	0.0287 (10)	0.0270 (8)	0.0029 (7)	0.0009 (6)	−0.0007 (7)
O3	0.0340 (8)	0.0391 (10)	0.0257 (7)	0.0029 (8)	0.0014 (6)	0.0036 (7)
O4	0.0506 (11)	0.0277 (10)	0.0382 (9)	0.0118 (9)	−0.0026 (7)	−0.0040 (7)
O5	0.0257 (8)	0.0284 (10)	0.0397 (9)	0.0018 (7)	0.0072 (6)	0.0026 (7)
C1	0.0646 (18)	0.0344 (16)	0.0288 (13)	0.0156 (14)	0.0024 (11)	−0.0013 (11)
C2	0.0313 (13)	0.0317 (14)	0.0262 (11)	0.0024 (11)	0.0055 (9)	0.0001 (10)
C3	0.0256 (11)	0.0299 (14)	0.0263 (11)	−0.0014 (10)	0.0041 (8)	−0.0009 (10)
C4	0.0264 (12)	0.0259 (13)	0.0333 (12)	−0.0015 (11)	0.0022 (9)	−0.0001 (10)
C5	0.0225 (11)	0.0265 (13)	0.0336 (12)	−0.0063 (10)	0.0030 (9)	−0.0040 (10)
C6	0.0273 (12)	0.0346 (14)	0.0312 (12)	−0.0036 (11)	−0.0020 (9)	−0.0030 (11)
C9	0.0378 (13)	0.066 (2)	0.0357 (13)	0.0138 (15)	0.0003 (10)	−0.0026 (13)
C10	0.144 (3)	0.050 (2)	0.0422 (16)	0.048 (2)	0.0237 (18)	0.0006 (14)
C11	0.0359 (13)	0.0372 (16)	0.0341 (12)	0.0030 (12)	−0.0020 (10)	−0.0041 (11)
C12	0.0444 (15)	0.0501 (18)	0.0364 (13)	0.0080 (14)	0.0019 (10)	−0.0003 (12)
C13	0.0500 (17)	0.067 (2)	0.0445 (15)	0.0013 (16)	0.0104 (12)	−0.0051 (15)
C14	0.0630 (18)	0.087 (3)	0.0279 (13)	−0.006 (2)	−0.0005 (12)	0.0018 (15)
C15	0.0548 (18)	0.077 (2)	0.0411 (16)	0.0043 (18)	−0.0142 (13)	0.0063 (16)
C16	0.0409 (15)	0.0557 (18)	0.0421 (15)	0.0055 (14)	−0.0052 (11)	−0.0024 (13)
C21	0.0590 (17)	0.0325 (16)	0.0403 (13)	0.0145 (14)	0.0118 (12)	−0.0024 (12)
C22	0.0435 (16)	0.057 (2)	0.0626 (19)	0.0016 (16)	−0.0069 (13)	−0.0192 (16)
C23	0.062 (2)	0.064 (2)	0.073 (2)	−0.0005 (18)	0.0332 (17)	0.0043 (19)
C24	0.078 (2)	0.088 (3)	0.0422 (16)	0.030 (2)	0.0101 (16)	0.0052 (17)
C25	0.0581 (19)	0.088 (3)	0.062 (2)	0.0231 (19)	−0.0177 (15)	−0.025 (2)
C26	0.0460 (17)	0.0468 (18)	0.074 (2)	−0.0019 (15)	0.0154 (14)	−0.0077 (16)

### Geometric parameters (Å, °)

**Table:**

O1—C1	1.421 (3)	C10—C21	1.498 (4)
O1—C10	1.433 (4)	C10—H10A	0.9900
O2—C2	1.431 (3)	C10—H10B	0.9900
O2—C6	1.440 (3)	C11—C16	1.381 (3)
O3—C9	1.413 (3)	C11—C12	1.393 (3)
O3—C3	1.429 (2)	C12—C13	1.379 (3)
O4—C4	1.426 (3)	C12—H12A	0.9500
O4—H4	0.8400	C13—C14	1.376 (4)
O5—C5	1.437 (2)	C13—H13A	0.9500
O5—H5	0.8400	C14—C15	1.382 (4)
C1—C2	1.505 (3)	C14—H14A	0.9500
C1—H1A	0.9900	C15—C16	1.381 (3)
C1—H1B	0.9900	C15—H15A	0.9500
C2—C3	1.531 (3)	C16—H16A	0.9500
C2—H2A	1.0000	C21—C26	1.381 (4)
C3—C4	1.526 (3)	C21—C22	1.390 (4)
C3—H3A	1.0000	C22—C23	1.381 (4)
C4—C5	1.518 (3)	C22—H22A	0.9500
C4—H4A	1.0000	C23—C24	1.355 (5)
C5—C6	1.511 (3)	C23—H23A	0.9500
C5—H5A	1.0000	C24—C25	1.355 (5)
C6—H6A	0.9900	C24—H24A	0.9500
C6—H6B	0.9900	C25—C26	1.372 (4)
C9—C11	1.503 (3)	C25—H25A	0.9500
C9—H9A	0.9900	C26—H26A	0.9500
C9—H9B	0.9900		
			
C1—O1—C10	113.0 (2)	C11—C9—H9B	109.6
C2—O2—C6	111.27 (16)	H9A—C9—H9B	108.1
C9—O3—C3	114.99 (16)	O1—C10—C21	112.9 (3)
C4—O4—H4	109.5	O1—C10—H10A	109.0
C5—O5—H5	109.5	C21—C10—H10A	109.0
O1—C1—C2	107.9 (2)	O1—C10—H10B	109.0
O1—C1—H1A	110.1	C21—C10—H10B	109.0
C2—C1—H1A	110.1	H10A—C10—H10B	107.8
O1—C1—H1B	110.1	C16—C11—C12	118.5 (2)
C2—C1—H1B	110.1	C16—C11—C9	119.1 (2)
H1A—C1—H1B	108.4	C12—C11—C9	122.4 (2)
O2—C2—C1	108.3 (2)	C13—C12—C11	120.5 (2)
O2—C2—C3	109.75 (16)	C13—C12—H12A	119.7
C1—C2—C3	112.95 (19)	C11—C12—H12A	119.7
O2—C2—H2A	108.6	C14—C13—C12	120.1 (3)
C1—C2—H2A	108.6	C14—C13—H13A	119.9
C3—C2—H2A	108.6	C12—C13—H13A	119.9
O3—C3—C4	111.07 (18)	C13—C14—C15	120.1 (2)
O3—C3—C2	107.03 (16)	C13—C14—H14A	119.9
C4—C3—C2	109.23 (17)	C15—C14—H14A	119.9
O3—C3—H3A	109.8	C16—C15—C14	119.5 (3)
C4—C3—H3A	109.8	C16—C15—H15A	120.2
C2—C3—H3A	109.8	C14—C15—H15A	120.2
O4—C4—C5	112.53 (17)	C15—C16—C11	121.2 (3)
O4—C4—C3	107.68 (17)	C15—C16—H16A	119.4
C5—C4—C3	111.47 (19)	C11—C16—H16A	119.4
O4—C4—H4A	108.3	C26—C21—C22	117.3 (3)
C5—C4—H4A	108.3	C26—C21—C10	120.6 (3)
C3—C4—H4A	108.3	C22—C21—C10	122.1 (3)
O5—C5—C6	108.29 (18)	C23—C22—C21	120.6 (3)
O5—C5—C4	111.39 (17)	C23—C22—H22A	119.7
C6—C5—C4	109.87 (17)	C21—C22—H22A	119.7
O5—C5—H5A	109.1	C24—C23—C22	120.2 (3)
C6—C5—H5A	109.1	C24—C23—H23A	119.9
C4—C5—H5A	109.1	C22—C23—H23A	119.9
O2—C6—C5	111.32 (16)	C25—C24—C23	120.4 (3)
O2—C6—H6A	109.4	C25—C24—H24A	119.8
C5—C6—H6A	109.4	C23—C24—H24A	119.8
O2—C6—H6B	109.4	C24—C25—C26	120.0 (3)
C5—C6—H6B	109.4	C24—C25—H25A	120.0
H6A—C6—H6B	108.0	C26—C25—H25A	120.0
O3—C9—C11	110.49 (18)	C25—C26—C21	121.6 (3)
O3—C9—H9A	109.6	C25—C26—H26A	119.2
C11—C9—H9A	109.6	C21—C26—H26A	119.2
O3—C9—H9B	109.6		
			
C10—O1—C1—C2	173.3 (2)	C3—O3—C9—C11	166.6 (2)
C6—O2—C2—C1	−173.07 (19)	C1—O1—C10—C21	−66.9 (4)
C6—O2—C2—C3	63.2 (2)	O3—C9—C11—C16	155.4 (3)
O1—C1—C2—O2	70.8 (2)	O3—C9—C11—C12	−26.4 (4)
O1—C1—C2—C3	−167.49 (18)	C16—C11—C12—C13	0.3 (4)
C9—O3—C3—C4	102.6 (2)	C9—C11—C12—C13	−177.8 (3)
C9—O3—C3—C2	−138.2 (2)	C11—C12—C13—C14	0.2 (5)
O2—C2—C3—O3	−178.27 (18)	C12—C13—C14—C15	−0.7 (5)
C1—C2—C3—O3	60.8 (2)	C13—C14—C15—C16	0.6 (5)
O2—C2—C3—C4	−57.9 (2)	C14—C15—C16—C11	0.0 (5)
C1—C2—C3—C4	−178.85 (19)	C12—C11—C16—C15	−0.4 (4)
O3—C3—C4—O4	−65.4 (2)	C9—C11—C16—C15	177.8 (3)
C2—C3—C4—O4	176.82 (17)	O1—C10—C21—C26	122.6 (3)
O3—C3—C4—C5	170.75 (16)	O1—C10—C21—C22	−58.1 (4)
C2—C3—C4—C5	52.9 (2)	C26—C21—C22—C23	1.1 (4)
O4—C4—C5—O5	−52.7 (2)	C10—C21—C22—C23	−178.3 (3)
C3—C4—C5—O5	68.4 (2)	C21—C22—C23—C24	0.2 (5)
O4—C4—C5—C6	−172.71 (18)	C22—C23—C24—C25	−0.5 (5)
C3—C4—C5—C6	−51.6 (2)	C23—C24—C25—C26	−0.4 (5)
C2—O2—C6—C5	−62.4 (2)	C24—C25—C26—C21	1.7 (5)
O5—C5—C6—O2	−66.5 (2)	C22—C21—C26—C25	−2.0 (4)
C4—C5—C6—O2	55.3 (2)	C10—C21—C26—C25	177.3 (3)

### Hydrogen-bond geometry (Å, °)

**Table:**

*D*—H···*A*	*D*—H	H···*A*	*D*···*A*	*D*—H···*A*
O4—H4···O5^i^	0.84	1.95	2.789 (2)	175
O5—H5···O2^i^	0.84	1.98	2.812 (2)	169
C1—H1A···O3	0.99	2.50	2.893 (3)	103
C6—H6B···O5^ii^	0.99	2.54	3.461 (3)	155

Symmetry codes: (i) −*x*+2, *y*−1/2, −*z*+1; (ii) *x*−1, *y*, *z*.
